# Simplified treatment protocols improve recovery of children with severe acute malnutrition in South Sudan: results from a mixed methods study

**DOI:** 10.1186/s41043-024-00518-2

**Published:** 2024-02-02

**Authors:** Emily Lyles, Sule Ismail, Maya Ramaswamy, Aly Drame, Eva Leidman, Shannon Doocy

**Affiliations:** 1 Johns Hopkins Bloomberg School of Public Health, 615 N Wolfe St, Suite E8132, Baltimore, MD 21205 USA; 2 Integral Global Consulting, Atlanta, GA USA; 3 US Centers for Disease Control and Prevention, Atlanta, GA USA

**Keywords:** Simplified nutrition treatment protocol, COVID-19 nutrition adaptations, Acute malnutrition, Community management of acute malnutrition, South Sudan

## Abstract

**Background:**

As part of COVID-19 mitigation strategies, emergency nutrition program adaptations were implemented, but evidence of the effects is limited. Compared to the standard protocol, the full adapted protocol included adapted admissions criteria, simplified dosing, and reduced visit frequency; partially adapted protocols consisting of only some of these modifications were also implemented. To enable evidence-based nutrition program modifications as the context evolved, this study was conducted to characterize how protocol adaptations in South Sudan affected Outpatient Therapeutic Feeding Program outcomes.

**Methods:**

A mixed methods approach consisting of secondary analysis of individual-level nutrition program data and key informant interviews was used. Analyses focused on program implementation and severe acute malnutrition treatment outcomes under the standard, full COVID-19 adapted, and partially adapted treatment protocols from 2019 through 2021. Analyses compared characteristics and outcomes by different admission types under the standard protocol and across four different treatment protocols. Regression models evaluated the odds of recovery and mean length of stay (LoS) under the four protocols.

**Results:**

Very few (1.6%; *n* = 156) children admitted based on low weight-for-height alone under the standard protocol would not have been eligible for admission under the adapted protocol. Compared to the full standard protocol, the partially adapted (admission only) and partially adapted (admission and dosing) protocols had lower LoS of 28.4 days (CI − 30.2, − 26.5) and 5.1 days (CI − 6.2, − 4.0); the full adapted protocol had a decrease of 3.0 (CI − 5.1, − 1.0) days. All adapted protocols had significantly increased adjusted odds ratios (AOR) for recovery compared to the full standard protocol: partially adapted (admission only) AOR = 2.56 (CI 2.18–3.01); partially adapted (admission + dosing) AOR = 1.78 (CI 1.45–2.19); and fully adapted protocol AOR = 2.41 (CI 1.69–3.45).

**Conclusions:**

This study provides evidence that few children were excluded when weight-for-height criteria were suspended. LoS was shortest when only MUAC was used for entry/exit but dosing and visit frequency were unchanged. Significantly shorter LoS with simplified dosing and visit frequency vs. under the standard protocol indicate that protocol adaptations may lead to shorter recovery and program enrollment times. Findings also suggest that good recovery is achievable with reduced visit frequency and simplified dosing.

## Background

Acute malnutrition persists as an important global health challenge that affected 45 million children under five years of age globally in 2022 [1]. The COVID-19 pandemic exacerbated the problem, leading to an estimated 9.3 million additional children suffering from acute malnutrition due to the economic impacts of pandemic restrictions, declines in food security, and reductions in health service availability [2]. Within the context of South Sudan, the COVID-19 pandemic coincided with conflict and climate shocks, leading to the most extreme levels of food insecurity since the country’s independence in 2011, with 8 million people facing extreme hunger in 2022 [3]. South Sudan’s global acute malnutrition (GAM) rate was estimated at 11.3% in 2022, and more than 1.3 million children were at risk of acute malnutrition in 2022 [4]. While children with acute malnutrition face increased morbidity and mortality risks [5], the condition is readily curable; however, as a means of reducing COVID-19 transmission risk, modifications were made to acute malnutrition treatment protocols in the early stages of the pandemic. Unfortunately, many of these modifications, implemented rapidly on a global scale, did not benefit from a strong body of evidence, and questions persist as to how protocol adaptations have impacted the treatment and recovery of children with acute malnutrition.

Community Management of Acute Malnutrition (CMAM) programs are implemented globally in settings with large caseloads of acutely malnourished children [6, 7]. Children with uncomplicated severe acute malnutrition (SAM) are most often treated in Outpatient Therapeutic Feeding Programs (OTP), where they are routinely monitored and provided with ready-to-use therapeutic food (RUTF) for consumption at home. Although most of the care is provided on an outpatient basis, the model necessitates close contact between health providers, children, and caregivers. A number of “simplified approaches” or adaptations to protocols for the management of acute malnutrition are commonly implemented “to improve effectiveness, quality, coverage and reduce the costs of caring for children with uncomplicated wasting” [8].

Under standard treatment guidelines, admission for nutrition programs is based on both mid-upper arm circumference (MUAC) and weight-for-height (WHZ), though the merits of this criteria are debated [9–11]. In light of concerns about physical contact during the COVID-19 pandemic, WHZ admission criteria were dropped in favor of MUAC or edema-only criteria. Additionally, in recent years there has been a move to simplify dosing of RUTF rations (historically given based on tables) to two RUTF packets per day for children with SAM [12–14]. In many programs, weekly follow-up is common, though there are emerging questions on whether less frequent follow-up might be as effective [15]. Finally, following recent wider efforts to decentralize CMAM programs and engage more community-level staff and structures, the World Health Organization (WHO) guidelines updated in 2023 now recommend community-level screening, management, and referral of children with uncomplicated MAM/SAM by trained and supervised community health workers [11, 16–18].

In line with Global Nutrition Cluster guidance [19], the South Sudan Ministry of Health and the South Sudan Nutrition Cluster revised national guidelines to ensure program continuity during the COVID-19 pandemic [19]. The recommended revisions adopted in April 2020 included: (1) admission based only on MUAC or edema; (2) fixed dosing of therapeutic foods, with two sachets daily for children with SAM instead of weight-based dosing under the standard protocol; (3) reduced follow-up frequency from weekly under the standard protocol to bi-weekly for SAM cases; and (4) use of community-based activities (e.g., provision of treatment by community health workers) where possible. Guidance was again updated on August 12, 2021, to include the need for a child to meet MUAC-based discharged criteria for two consecutive visits prior to being discharged as cured [20]. OTP follow-up frequency was returned to weekly in August 2021, but other mitigation measures remained in place through the end of 2021 [20].

Evidence reviews suggest that simplified acute malnutrition treatment protocols can contribute to an increase in the number of children treated due to greater efficiency of the modified dosing regimen, and that treatment quality remains high and exceeds international Sphere standards [8–10, 12–18, 21–25]. Nevertheless, the overall volume and strength of the evidence is limited—notably, there is a paucity of studies that examine individual-level data at scale. The available ecological analyses cannot differentiate whether changes were due to revised treatment protocols or changes in the population structure that resulted from revised MUAC-only admission criteria, where children previously qualified based on WHZ alone are no longer eligible. As such, questions remain about the simultaneous adoption of different protocol changes at scale in a non-research setting that are best addressed with an individual-level analysis of program participants. To enable evidence-based nutrition program modifications as the context of South Sudan evolved, we undertook a mixed methods study to characterize how protocol adaptations affected OTP outcomes in South Sudan. To inform future nutrition policy in South Sudan, the study’s main objectives were to compare SAM treatment outcomes under the standard, full adapted, and partially adapted treatment protocols [controlling for child age, sex, and nutrition status at program entry], to understand differences in program implementation under the various protocols, and to examine how MUAC-only admission criteria shape the population of SAM children eligible for OTP under the standard treatment protocol.

## Methods

A mixed methods approach consisting of secondary analysis of individual-level non-governmental organizations’ (NGOs) CMAM program data and key informant interviews (KIIs) was used. The analysis focused on OTP implementation and SAM treatment outcomes under the standard, full COVID-19 adapted, and partially adapted treatment protocols from 2019 through 2021.

### Secondary analysis of program data

**Fig. 1:**
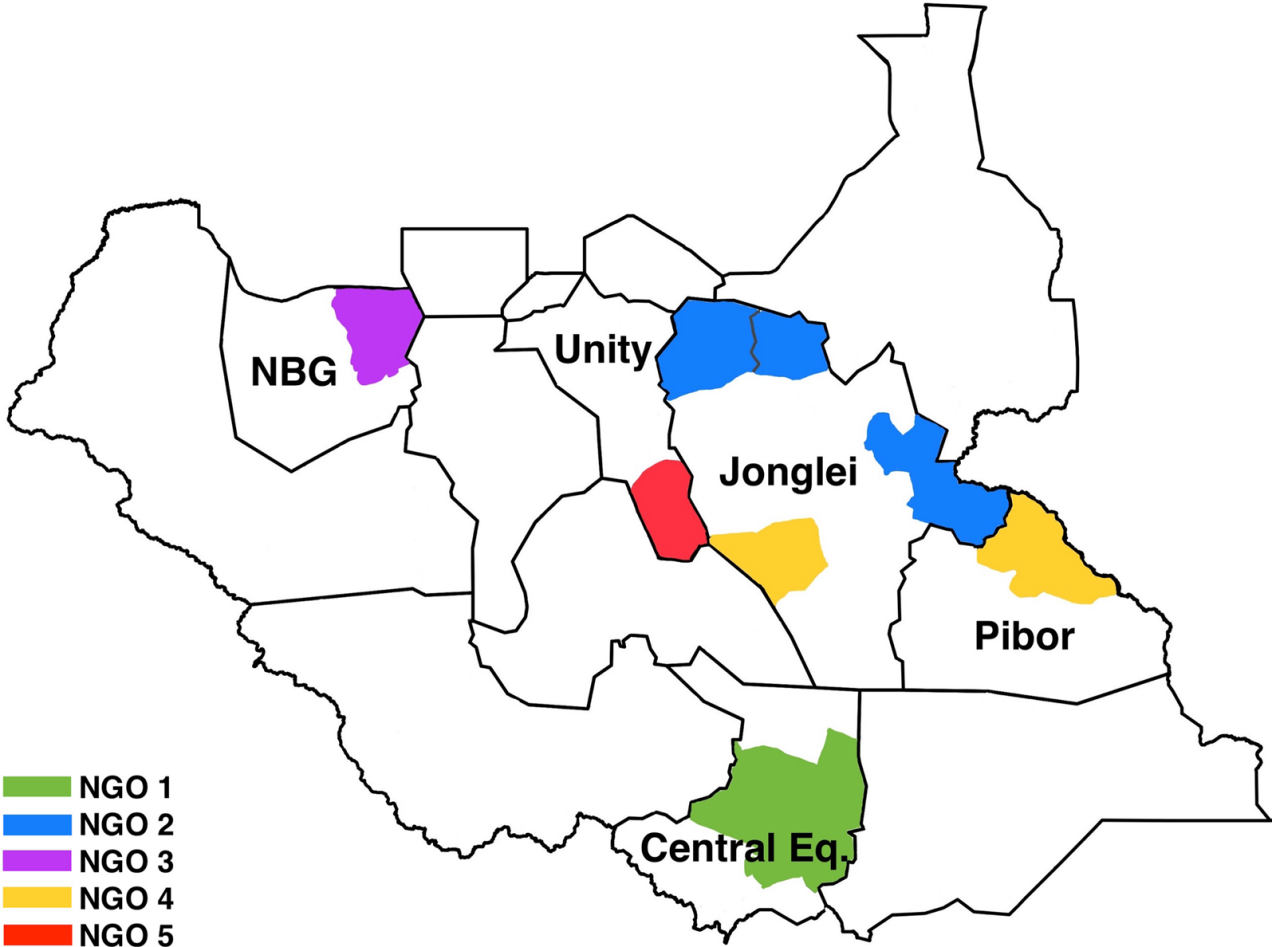
Map of counties with CMAM data available

**Fig. 2:**
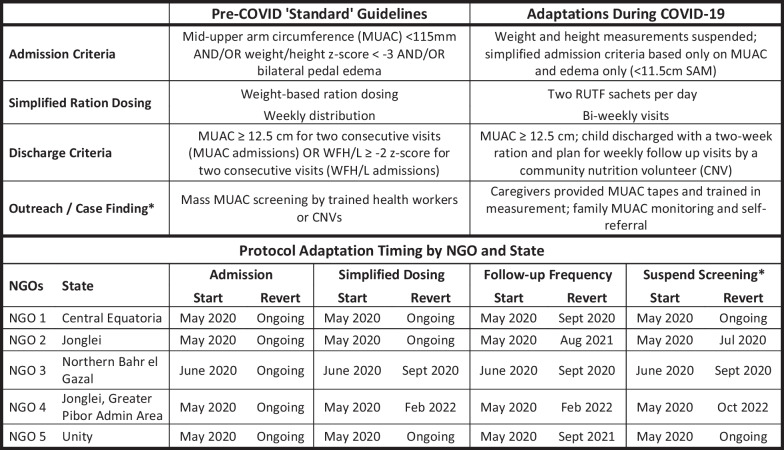
South Sudan guideline adaptations for outpatient therapeutic feeding programs in response to COVID-19

A standard component of nutrition program management is the recording of individual data (age, sex, anthropometric measurements, date) at admission and discharge. To better understand how nutrition protocol adaptations affected program performance, all NGOs in the nutrition cluster were invited to share these de-identified child-level data. Five NGOs responded and shared a total of 83,618 records from treatment facilities in four different states (Central Equatoria, Lakes, Northern Bahr el Ghazal, and Unity) (Fig. 1). As modifications to CMAM treatment protocols were not implemented by all partners or in all geographic areas at the same time, an overview of CMAM treatment guideline adaptations in response to COVID-19 in South Sudan is provided in Fig. 2, along with each adaptation’s implementation dates for the five NGOs from which CMAM data were received. Records were from January 2019–December 2021 and were provided either as a database export or entered by NGOs into a standardized Excel template (if record keeping was done in paper format).

Datasets were merged, and all data were cleaned prior to analysis to check for duplicates, outliers, and missing data. Anthropometric outliers among children enrolled in OTP were not dropped from the dataset; however, length of stay (LoS) values greater than 120 days (89th percentile for SAM children) were considered outliers and dropped, given the overall distribution and plausibility in the context. Each record was categorized as occurring under four different treatment protocol categories: (1) standard protocol, (2) full adapted protocol [adapted admissions, dosing, and follow-up frequency], (3) partially adapted protocol [adapted admissions only], and (4) partially adapted protocol [adapted admissions and dosing] based on the admission date. Each protocol category’s date ranges were defined independently for each NGO, given differences in the timing of adopting protocol changes.

All quantitative analyses were performed using STATA 15 (StataCorp, USA). The main outcomes of interest were recovery and LoS. Descriptive analyses also examined children’s sex and age, average MUAC at admission, change in MUAC from admission to discharge, and exit outcomes (cured/recovered, deceased, defaulted, non-responsive, and transferred). Differences in descriptive statistics across the four treatment protocols were examined using chi-square and t test methods for binary/categorical and continuous variables, respectively. A similar analysis was undertaken for different admission types (i.e., WHZ only, MUAC only, WHZ and MUAC) under the standard protocol to characterize how the MUAC-only admission criteria impacted the age and sex composition of enrolled children. Regression models were used to evaluate the odds of recovery (logistic regression) and mean LoS (linear regression) for children enrolled in 2021 under the adapted protocols as compared to children enrolled in 2019 under the standard protocol (reference group); 2020 enrollments were excluded from the models to allow for protocol comparison during periods of greater stability where fewer exogenous factors impacted program implementation. Unadjusted models were first fit to evaluate the crude effect of each treatment protocol on the outcomes of interest (recovery and LoS). Adjusted estimates were then obtained using mixed effects models with NGO included as a random effect and protocol type, child age (continuous), sex, and MUAC at admission (continuous) as fixed effects.

### Key informant interviews

Group KIIs were conducted using a semi-structured questionnaire with content focused on each of the treatment protocol adaptations (admissions/exit criteria, simplified dosing, reduced visit frequency, suspension of case finding), challenges with implementation and impacts in terms of service delivery and program outcomes. All NGO members of the South Sudan Nutrition Cluster with active CMAM programs were invited to participate. For each organization interested in participating, the senior-level nutrition/health staff was requested to identify a Juba-based nutrition staff and a senior nutrition staff from each state with CMAM programming. Interviews with staff from each NGO were conducted virtually after obtaining verbal consent; the semi-structured group interviews lasted 60–90 min and were conducted in English by a trained facilitator that was independent and a member of the JHU/CDC research team. Interviews were also audio-recorded to confirm the completeness and accuracy of transcripts. Transcripts were reviewed independently to develop a list of codes, which were then compared for consistency and merged into a codebook in MAXQDA (Verbi Software, Germany). Each transcript was coded by two team members and reviewed jointly for consistency and to address discrepancies; reports were then generated by codes and sub-codes. Coded segments were used to identify key findings consistent across multiple respondents, predefined themes, or related to factors that impacted program outcomes [26].

### Ethical review

The study was reviewed and approved by the South Sudan Ministry of Health Ethics Committee and Institutional Review Board at Johns Hopkins Bloomberg School of Public Health. This activity was reviewed by the US Centers for Disease Control and Prevention (CDC) and was conducted consistent with applicable federal laws and CDC policy.

## Results

**Fig. 3:**
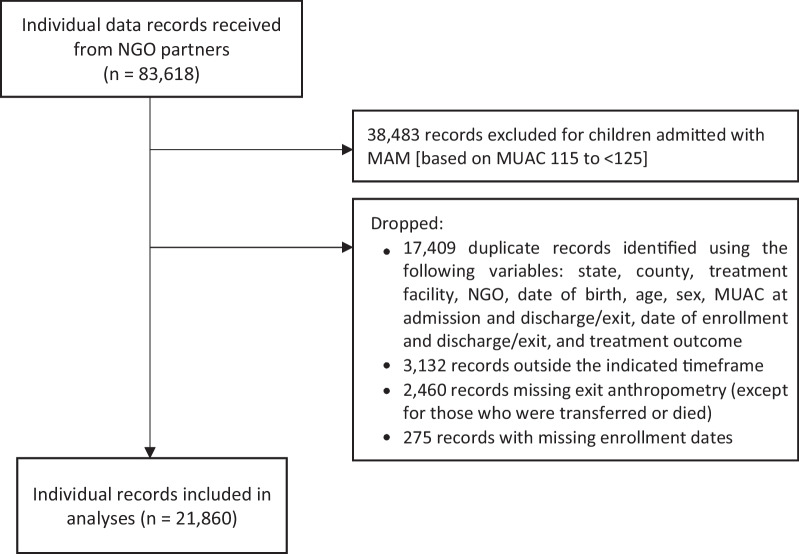
Data cleaning flow diagram

Of the 83,618 individual data records provided by NGO partners, secondary data analysis included records from 21,860 children enrolled in OTP at 61 CMAM sites (see data cleaning flow chart in Fig. 3). The numbers of CMAM sites and records included in the analysis are presented in Table 1, disaggregated by NGO, state, and protocol. Half of the records were from sites in Jonglei State, and a third were from Northern Bahr el Ghazal; the remainder were from Central Equatoria, the Greater Pibor Administrative Area, and Unity State each contributed 4–8% of cases. Similarly, two NGOs contributed approximately three-quarters of the sample, with the other three NGOs collectively contributing the remaining quarter of cases. Nearly half of the analyzed records occurred under the standard protocol, while similar proportions (15–20%) were under the fully adapted (admission, dosing, reduced visit frequency), partially adapted (admission and dosing), and partially adapted (admission only) protocols. Qualitative data were collected from 34 key informants (2–4 per NGO) representing 10 NGOs (8 international, 2 national) in 10 group interviews (one per organization). Collectively, these organizations oversee 566 outpatient CMAM program sites and 34 inpatient stabilization centers across all states and administrative areas in South Sudan. All except one NGO that provided individual-level data participated in the KIIs.

**Table 1:** OTP Admissions by NGO, location, and treatment protocol (*n* = 21,860)

By NGO service provider				By location				By treatment protocol				
NGO	# of Sites	# of Records		State	# of Sites	# of Records		Protocol	Timeframe	# of Sites	# of Records	
		*N*	%			*N*	%				*N*	%
NGO 1	11	1706	7.8	Central Equatoria	11	1706	7.8	Standard Protocol	Jan 2019–Apr 2020	58	10,006	45.8
NGO 2	22	9279	42.4	Jonglei	28	10,960	50.1	Fully Adapted Protocol	May 2020-Dec 2021	34	3436	15.7
NGO 3	17	7302	33.4	Northern Bahr el Ghazal (NBG)	17	7302	33.4	Partially Adapted: Admission + Dosing	Aug 2020-Dec 2021	28	4128	18.9
NGO 4	9	2734	12.5	Pibor Admin. Area	3	1053	4.8	Partially Adapted: Admission Only	Sep 2020-Dec 221	17	4290	19.6
NGO 5	2	839	3.8	Unity	2	839	3.8					

### Characteristics of the CMAM eligible population by admission criteria

To address questions related to how MUAC-only admission criteria shape the population of SAM children eligible for OTP, we analyzed children enrolled under the standard protocol where children are admitted based on low WHZ only, low MUAC only, and both low MUAC and WHZ (compared to the adapted protocol under which children are admitted based only on low MUAC [weight and height are not measured]). Differences in SAM children enrolled under the standard treatment protocol based on admission criteria are presented in Table 2. A total of 1.6% of children (*n* = 156) would not have been eligible for OTP if the adapted protocol was in place because they qualified based on WHZ alone.

**Table 2:** Descriptive analysis of OTP admissions and treatment outcomes under the standard protocol by admission type

	Overall	WHZ only	MUAC only	MUAC + WHZ
# of sites	58	26	47	25
# of records	*N* = 10,006	*n* = 156	*n* = 7263	*n* = 2587

	Point (95 CI)	Point (95 CI)	Point (95 CI)	Point (95 CI)	*p*-value
Age at enrollment (months)	*n* = 9936	*n* = 155	*n* = 7200	*n* = 2581	
Median (range)	15 (1.0–59.0)	14 (6.0–48.0)	13 (1.0–59.0)	23 (6.0–58.0)	< 0.001
Mean (CI)	18.1 (17.9–18.3)	18.4 (16.7–20.1)	16.2 (16.0–16.4)	23.4 (22.9–23.9)	
6–12 months	42.5% (41.6–43.5%)	36.8% (29.1–44.5%)	46.3% (45.1–47.4%)	32.4% (30.6–34.2%)	< 0.001
13–24 months	35.8% (34.9–36.7%)	43.9% (36.0–51.8%)	39.2% (38.1–40.3%)	25.8% (24.1–27.5%)	
25–36 months	16.2% (15.5–16.9%)	12.3% (7.0–17.5%)	11.7% (10.9–12.4%)	29.1% (27.3–30.8%)	
37–48 months	4.5% (4.1–4.9%)	7.1% (3.0–11.2%)	2.3% (2.0–2.7%)	10.2% (9.1–11.4%)	
49–59 months	1.0% (0.8–1.2%)	0.0% (0.0–0.0%)	0.5% (0.3–0.7%)	2.5% (1.9–3.1%)	
Sex	*n* = 10,003	*n* = 156	*n* = 7261	*n* = 2586	
Female	54.9% (53.9–55.8%)	42.3% (34.5–50.1%)	56.9% (55.8–58.1%)	49.8% (47.9–51.7%)	< 0.001
Male	45.1% (44.2–46.1%)	57.7% (49.9–65.5%)	43.1% (41.9–44.2%)	50.2% (48.3–52.1%)	
MUAC at enrollment (mm)	*n* = 9961	*n* = 111	*n* = 7263	*n* = 2587	
Median (range)	112 (70.0–125.0)	117 (115.0–125.0)	112 (70.0–114.0)	112 (86.0–114.0)	< 0.001
Mean (CI)	111.0 (110.9–111.0)	117.7 (117.2–118.3)	110.8 (110.7–110.9)	111.2 (111.1–111.3)	
Change in MUAC from enrollment to discharge (mm)	*n* = 9954	*n* = 111	*n* = 7256	*n* = 2587	
Median (range)	6 (− 43.0–46.0)	1 (− 21.0–11.0)	6 (− 43.0–46.0)	6 (− 17.0–30.0)	< 0.001
Mean (CI)	6.4 (6.3–6.4)	0.2 (− 0.8–1.1)	6.4 (6.3–6.5)	6.6 (6.4–6.7)	
Length of stay	*n* = 7332	*n* = 153	*n* = 4626	*n* = 2553	
Median (IQR)	53 (36.0–70.0)	42 (0.0–114.0)	50 (0.0–120.0)	56 (0.0–120.0)	< 0.001
Mean (CI)	55.3 (54.8–55.9)	41.1 (37.3–44.8)	56.1 (55.3–56.9)	54.8 (54.1–55.5)	
Exit type	*n* = 10,006	*n* = 156	*n* = 7263	*n* = 2587	
Cured	88.0% (87.4–88.7%)	56.4% (48.5–64.3%)	88.8% (88.1–89.5%)	87.8% (86.5–89.0%)	< 0.001
Default	2.5% (2.2–2.8%)	2.6% (0.1–5.1%)	2.1% (1.8–2.5%)	3.5% (2.8–4.2%)	
Death	0.0% (0.0–0.1%)	0.0% (0.0–0.0%)	0.1% (0.0–0.1%)	0.0% (0.0–0.0%)	
Non-response	2.5% (2.2–2.8%)	0.0% (0.0–0.0%)	2.9% (2.5–3.3%)	1.6% (1.1–2.1%)	
Not recovered/unknown	4.4% (4.0–4.8%)	35.3% (27.7–42.8%)	2.8% (2.5–3.2%)	7.0% (6.0–8.0%)	
Transfer	2.5% (2.2–2.8%)	5.8% (2.1–9.5%)	3.3% (2.9–3.7%)	0.2% (0.0–0.3%)	

*CI* Confidence interval, *IQR* Interquartile range, *MUAC* Mid-upper arm circumference, *WHZ* Weight-for-height *z*-score

Children admitted based only on WHZ had a mean age of 18.4 months, similar to the total population (18.1 months). Children that qualified based only on MUAC had the lowest mean age (16.2 months), whereas those meeting both WHZ and MUAC admission criteria were significantly older (23.4 months, *p* < 0.001). Fewer female children were admitted based on WHZ only (42.3%) compared to WHZ + MUAC (49.8%) and MUAC only (56.9%) (*p* < 0.001). Children admitted based only on WHZ had the highest average MUAC at enrollment (117.7 mm vs. 111.2 for WHZ + MUAC and 110.8 for MUAC only, *p* < 0.001) and the lowest average change in MUAC (0.2 mm vs. 6.6 for WHZ + MUAC only and 6.4 for MUAC only, *p* < 0.001). However, children admitted based on WHZ only also had the shortest average LoS (41.1 days vs. 54.8 for WHZ + MUAC and 56.1 for MUAC only, *p* < 0.001) and the lowest recovery rate of all three groups (56.4% vs. 87.8% for WHZ + MUAC and 88.8% for MUAC only, *p* < 0.001). Of children eligible based on WHZ only, the proportion that died (2.6%) was similar to the other groups, but referrals to inpatient stabilization centers (5.8%) and unrecovered exits with unknown outcomes (35.3%) [including children that did not meet exit criteria but where no outcome (e.g., default, non-response, transfer) was reported] were both more frequent.

### OTP outcomes by protocol type

To characterize differences in recovery and LoS, we conducted a descriptive analysis of SAM admissions and treatment outcomes by protocol type (Table 3). The age of admitted children differed significantly across protocols, ranging from a mean age of 19.5 months (median 18) for the partially adapted (admission only) protocol to a mean age of 15.8 months (median 13) in children admitted under the partially adapted (admission and dosing) protocol. Given that the same admission criteria were applied across adapted protocols, this is likely a function of different geographies and demographics. All protocols had similar sex distributions, with slightly more females than males admitted (55% vs 45%). Mean MUAC at enrollment was relatively similar for children under all protocols, but children admitted under the partially adapted (admission only) protocol had a significantly greater change in MUAC from enrollment to exit, averaging 7.0 mm compared to the smallest change of 5.4 mm under the fully adapted protocol (*p* < 0.001).

**Table 3:** Descriptive analysis of OTP admissions and treatment outcomes by protocol

	Overall	Full standard protocol	Partially adapted protocol: admission only	Partially adapted protocol: admission + dosing	Fully adapted protocol
# of sites	61	58	17	28	34
# of records	*N* = 21,860	*n* = 10,006	*n* = 4290	*n* = 4128	*n* = 3436

	Point (95 CI)	Point (95 CI)	Point (95 CI)	Point (95 CI)	Point (95 CI)	*p*-value
Age at enrollment (months)	*n* = 21,770	*n* = 9936	*n* = 4290	*n* = 4127	*n* = 3417	
Median (range)	15 (1.0–59.0)	15 (1.0–59.0)	18 (6.0–59.0)	13 (1.0–58.0)	14 (6.0–59.0)	< 0.001
Mean (CI)	17.8 (17.6–17.9)	18.1 (17.9–18.3)	19.5 (19.2–19.9)	15.8 (15.5–16.0)	17.1 (16.8–17.4)	
6–12 months	42.3% (41.7–43.0%)	42.5% (41.6–43.5%)	35.2% (33.8–36.6%)	47.5% (45.9–49.0%)	44.5% (42.8–46.2%)	< 0.001
13–24 months	37.5% (36.9–38.1%)	35.8% (34.9–36.7%)	38.8% (37.3–40.2%)	41.4% (39.8–42.9%)	36.2% (34.6–37.8%)	
25–36 months	15.2% (14.7–15.6%)	16.2% (15.5–16.9%)	19.3% (18.1–20.5%)	8.7% (7.8–9.6%)	14.7% (13.5–15.9%)	
37–48 months	4.3% (4.0–4.6%)	4.5% (4.1–4.9%)	5.9% (5.2–6.6%)	2.3% (1.8–2.8%)	4.2% (3.6–4.9%)	
49–59 months	0.7% (0.6–0.8%)	1.0% (0.8–1.2%)	0.8% (0.6–1.1%)	0.2% (0.0–0.3%)	0.4% (0.2–0.6%)	
Sex	*n* = 21,823	*n* = 10,003	*n* = 4289	*n* = 4117	*n* = 3414	
Female	54.8% (54.1–55.4%)	54.9% (53.9–55.8%)	54.3% (52.8–55.8%)	55.4% (53.8–56.9%)	54.3% (52.7–56.0%)	0.744
Male	45.2% (44.6–45.9%)	45.1% (44.2–46.1%)	45.7% (44.2–47.2%)	44.6% (43.1–46.2%)	45.7% (44.0–47.3%)	
MUAC at Enrollment (mm)	*n* = 21,812	*n* = 9961	*n* = 4288	*n* = 4128	*n* = 3435	
Median (range)	112 (70.0–135.0)	112 (70.0–125.0)	112 (71.0–135.0)	112 (83.0–114.0)	112 (84.0–114.0)	< 0.001
Mean (CI)	111.2 (111.1–111.2)	111.0 (110.9–111.0)	111.1 (111.0–111.3)	111.3 (111.2–111.4)	111.6 (111.5–111.7)	
Change in MUAC from Enrollment to Discharge (mm)	*n* = 21,798	*n* = 9954	*n* = 4284	*n* = 4125	*n* = 3435	
Median (range)	6 (− 43.0–46.0)	6 (− 43.0–46.0)	7 (− 22.0–45.0)	5 (− 28.0–41.0)	5 (− 21.0–33.0)	< 0.001
Mean (CI)	6.3 (6.2–6.3)	6.4 (6.3–6.4)	7.0 (6.9–7.1)	6.1 (6.0–6.2)	5.4 (5.3–5.6)	
Length of stay	n = *18,370*	n = *7332*	n = *4263*	n = *3974*	n = *2801*	
Median (IQR)	49 (34.0–67.0)	53 (36.0–70.0)	42 (28.0–62.0)	43 (32.0–59.0)	47 (35.0–70.0)	< 0.001
Mean (CI)	51.0 (50.7–51.4)	55.3 (54.8–55.9)	45.3 (44.6–46.0)	48.0 (47.3–48.7)	52.7 (51.8–53.7)	
Time to recovery*	*n* = 16,757	*n* = 6569	*n* = 3677	*n* = 3839	*n* = 2672	
Median (IQR)	49 (35.0–64.0)	53 (37.0–70.0)	42 (28.0–58.0)	42 (32.0–58.0)	47 (35.0–70.0)	< 0.001
Mean (CI)	51.1 (50.7–51.4)	55.5 (54.9–56.1)	45.2 (44.5–45.9)	47.8 (47.1–48.5)	52.9 (51.9–53.8)	
Exit type	*n* = 21,860	*n* = 10,006	*n* = 4290	*n* = 4128	*n* = 3436	
Cured	90.3% (89.9–90.6%)	88.0% (87.4–88.7%)	85.9% (84.9–87.0%)	95.8% (95.2–96.4%)	95.5% (94.8–96.2%)	< 0.001
Default	1.8% (1.6–2.0%)	2.5% (2.2–2.8%)	2.2% (1.8–2.6%)	0.4% (0.2–0.5%)	1.1% (0.8–1.5%)	
Death	0.1% (0.0–0.1%)	0.0% (0.0–0.1%)	0.1% (0.0–0.3%)	0.0% (0.0–0.0%)	0.0% (0.0–0.0%)	
Non-response	2.2% (2.0–2.4%)	2.5% (2.2–2.8%)	4.0% (3.4–4.6%)	1.1% (0.8–1.5%)	0.3% (0.1–0.5%)	
Not recovered/ outcome unknown	3.2% (3.0–3.4%)	4.4% (4.0–4.8%)	1.6% (1.2–2.0%)	2.6% (2.1–3.1%)	2.4% (1.9–2.9%)	
Transfer	2.5% (2.3–2.7%)	2.5% (2.2–2.8%)	6.1% (5.4–6.8%)	0.1% (0.0–0.2%)	0.7% (0.4–1.0%)	

*CI* Confidence interval, *IQR* Interquartile range, *MUAC* Mid-upper arm circumference

*Mean length of stay among only children who exited as cured

### Length of stay

**Fig. 4:**
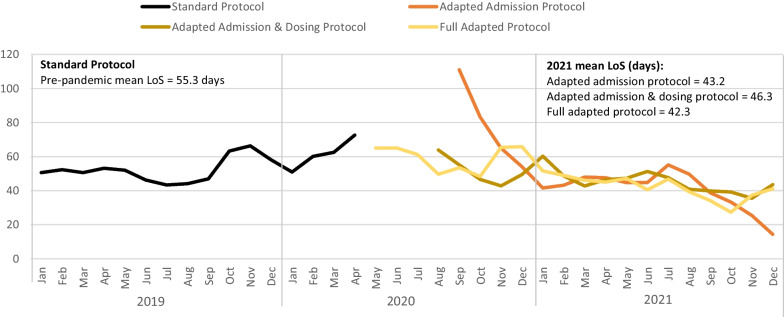
Mean length of OTP stay (in days) by protocol type and month/year

Mean LoS decreased for all adapted protocols over time after the onset of the pandemic (Fig. 4). The mean LoS for June to December 2020 was 57.6 compared to 41.0 for the same period in 2021 (*p* < 0.001). There were statistically significant differences in mean LoS across the four protocols, presented in Table 3; one potential explanation for shorter LoS during the pandemic period as compared to pre-COVID is changes in admission and exit criteria (MUAC only vs. MUAC or WHZ). To characterize protocol performance in a more stable period when NGOs were better adapted to pandemic operations, and there were fewer restrictions, LoS was compared for the three adapted protocols for January to December 2021. In this analysis, mean LOS under the fully adapted protocol (42.3 days) was significantly shorter than under the partially adapted (admission and dosing) protocol (46.3 days), but only slightly shorter than under the partially adapted (admission only) protocol (43.2 days), which suggests that reduced visit frequency may have significantly impacted LoS in the later stages of the pandemic. Nearly all key informants observed that children’s LoS increased with reduced visit frequency during COVID-19 (captured in our analysis under the fully adapted protocol). According to key informants, the change in the follow-up schedule led caretakers to forget and miss follow-up visits, thus requiring children to stay in the program for longer to complete the two consecutive visits necessary for discharge. As one key informant explained:

“The modified follow-up frequency affected the length of stay of beneficiaries in OTP and TSFP. So, the length of stay was increased, reason being, this child did not turn up for an appointment on a particular date because they forgot; at the end, a child expected to recover in 6 weeks, may end up recovering after, like 8 weeks, longer than really expected, because the caregivers forgot the days they were supposed to return. When they forget and come after some days, it means that there are days they will go without supplies, hence they deteriorate.” – NGO KII participant

It was also reported that caregivers would share or sell supplies during the longer follow-up period, causing children to go without therapeutic food for a time prior to the next visit. Simplified dosing was perceived as related to longer LoS and reduced recovery rates because older children received fewer RUTF sachets (two per day) compared to what they would have received with weight-based dosing.

All adapted protocol types were associated with decreased LoS compared to the full standard protocol (Table 4). Both the partially adapted (admission only) protocol and the partially adapted (admission and dosing) protocol had lower LoS, with decreases of 28.4 days (CI − 30.2, − 26.5) and 5.1 days (CI − 6.2, − 4.0); neither of these protocols included reduced visit frequency. The fully adapted protocol, which incorporated reduced visit frequency, had a decrease of 3.0 (CI − 5.1, − 1.0) days in LoS. Differences for most pairwise comparisons of adapted protocol types were also statistically significant (*p* < 0.001), except for the partially adapted (admission and dosing) and fully adapted protocols, which did not significantly differ from one another (*p* = 0.072). Child MUAC at admission was negatively associated with LoS, with a 0.77 day decrease in LoS (CI − 0.87, − 0.67) per mm increase in mean MUAC. Child age and sex were not significantly associated with LoS in adjusted models (*p* = 0.294 and *p* = 0.701, respectively).

**Table 4:** Unadjusted and adjusted mean length of stay and odds of recovery for OTP in 2019 and 2021 by child characteristics and protocol

	Mean length of stay (in days)			Odds of recovery		
	Point	(95% CI)	*p*-value	OR	(95% CI)	*p*-value
Unadjusted model						
Protocol type						
Full standard protocol	Reference group			Reference group		
Partially adapted: admission only	− 9.96	(− 10.91–9.01)	< 0.001	0.87	(0.77–0.97)	0.014
Partially adapted: admission + dosing	− 6.86	(− 7.89–5.82)	< 0.001	2.89	(2.41–3.47)	< 0.001
Full adapted protocol	− 10.86	(− 12.26–9.46)	< 0.001	4.02	(3.06–5.28)	< 0.001
Adjusted model^a^						
Protocol type						
Full standard protocol	Reference group			Reference group		
Partially adapted: admission only	− 28.35	(− 30.18–26.51)	< 0.001	2.56	(2.18–3.01)	< 0.001
Partially adapted: admission + dosing	− 5.09	(− 6.15–4.03)	< 0.001	1.78	(1.45–2.19)	< 0.001
Full adapted protocol	− 3.01	(− 5.05–0.98)	0.004	2.41	(1.69–3.45)	< 0.001
Age (months)	− 0.02	(− 0.06–0.02)	0.294	1.00	(1.00–1.01)	0.406
Female sex	− 0.15	(− 0.90–0.61)	0.701	1.12	(1.00–1.26)	0.042
MUAC at admission (in mm)	− 0.77	(− 0.87–0.67)	< 0.001	1.14	(1.13–1.15)	< 0.001
ICC (NGO)	0.1553			0.1143		
* N*	13,451			14,774		

*ICC* Intraclass correlation coefficient, *OR* Odds ratio

^a^Models also included NGO as a random effect

### Recovery Rate

**Fig. 5:**
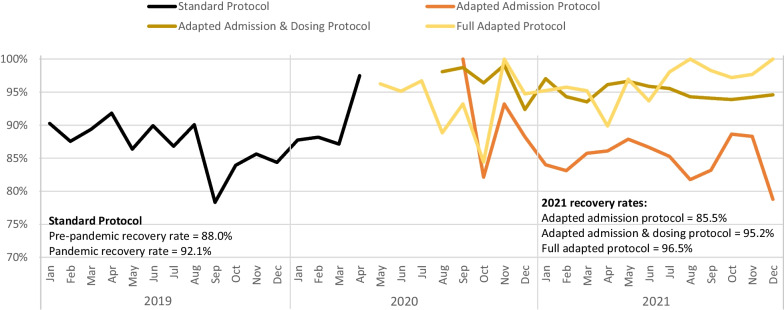
OTP recovery rate by protocol type and month/year

Recovery rates by protocol type and month/year are presented in Table 3 and Fig. 5. The pre-pandemic recovery rate was 88.0% compared to 92.1% during the pandemic (*p* < 0.001). During the pandemic, recovery rates were relatively volatile over time for the same protocol, but differences between protocols were more pronounced. The recovery rates in the first half of 2020 and the first half of 2021 were 63.3% and 47.5%, respectively (*p* < 0.001). During the pandemic, the highest recovery rates were seen under the partially adapted (admission and dosing) (95.8%) and fully adapted protocol (95.5%), while the partially adapted (admission only) (85.9%) protocols had notably lower recovery rate (*p* < 0.001). In the pandemic period, the partially adapted admission only protocol had the highest default (2.2%), non-response (4.0%), and transfer (6.1%) rates, but the mortality rate was low (0.1%) and similar to that of the other adapted protocols. In contrast, to the observed increase in recovery rate during the pandemic, KII participants reported higher non-response and default rates under adapted protocols, which they attributed to the reduction in commodities dispensed, supply misuse (including sharing and selling), supply chain shortages, and flood-related disruptions on the NGO side. Among the population receiving treatment, increased default and non-response rates were mostly attributed to caregivers forgetting their appointments due to changes in follow-up visit schedules, but fears of COVID-19 transmission, flooding, and insecurity were, among other factors, less frequently reported. Additionally, some key informants felt that default rates were more related to logistics and stock-outs of supplies than they were to protocol changes; as one participant explained:

“No, the defaulter [rate] is not related to the simplified protocol. The increasing defaulter rate is when the stock-out leads to logistic issue. Because of the flooding and because of donor factors, which is … they are not using [helicopters], they are using river transport, [which] takes one month … and during that period we may face stock-out of supply that is resulting in increasing in defaulter rate.” – NGO KII participant

Simplified dosing was not perceived as related to longer LoS; participants in qualitative interviews suggested the simplified dosing of RUTF, from weight-based to two sachets per child per day, led to longer LoS and reduced recovery rates for older children because they received fewer sachets than what would have previously been allocated with weight-based dosing. One key informant described this problem by saying:

“It affects the recovery, the energy that this child is supposed to have, the therapeutics, in particular the RUTF because you are only supposed to get that 2 sachets and it could not be enough to achieve the kilocalories for this child because you have made everything to be uniform whether [the child is] big or small, yet if [dosing] was [based on] weight, it could vary according to the Kg of the child and the [child] who weighs more equally gets more.” – NGO KII participant

Unadjusted models comparing odds of recovery by treatment protocol are presented in Table 4, along with mixed effects models estimating the adjusted odds ratios (AOR) for recovery under adapted protocols compared to the standard protocol. All adapted protocols had significantly increased AORs for recovery, as follows: partially adapted (admission only) AOR = 2.56 (CI 2.18–3.01); partially adapted (admission + dosing) AOR = 1.78 (CI 1.45–2.19); and fully adapted protocol AOR = 2.41 (CI 1.69–3.45). Differences in odds of recovery for all pairwise comparisons of adapted protocol types were also statistically significant (*p* < 0.001 for all). There were no significant differences in the AOR for recovery by child age (*p* = 0.406); however, both the female sex and MUAC were positively associated with recovery, with a 12% increase in recovery odds for female children (AOR = 1.12, CI 1.00–1.26) and a 14% increase in recovery odds per additional millimeter enrollment MUAC measure (AOR = 1.14, CI 1.13–1.15).

## Discussion

This study leveraged existing 2019 to 2021 CMAM program data to examine the impacts of nutrition treatment protocol changes in response to the COVID-19 pandemic in South Sudan. Application of modified MUAC-only admission criteria to the total population of children receiving treatment prior to the pandemic resulted in 1.4% of children being ineligible for treatment as they qualified based on weight-for-height alone. All three adapted protocols had shorter LoS than the standard treatment protocol, and mean LoS in OTP decreased during COVID-19. Recovery rates improved during COVID-19, in both adjusted and unadjusted analyses, suggesting that protocol modifications such as reduced visit frequency and simplified dosing were beneficial.

Interestingly, girls comprised 54.8% of CMAM program participants, despite global and national evidence that prevalence of wasting is higher among boys. In South Sudan, the male/female wasting prevalence ratio is estimated at 1.73 (CI 1.62–1.85); however, while girls in South Sudan have higher WHZ than boys, they have lower MUAC, which is a likely explanation for why they account for more than half of CMAM admissions [27, 28].

### Admission criteria

An analysis of health information system data from 17 countries in Africa observed a 14% decline in SAM admissions when comparing April to June of 2020 with the same period the preceding year. Despite the overall decline, countries experienced both increases and decreases in admissions, and changes are attributable to a variety of factors beyond COVID-19 [29]. A 21.8% decline in SAM admissions was observed during the COVID-19 pandemic in South Sudan [30]. A potential driver of this decline is modified MUAC-only admission criteria, where fewer children would be eligible if WHZ is dropped as an admission criteria. While this concern has been documented among policymakers in South Sudan [31], the operational benefits of the MUAC-only approach are significant. The MUAC and/or edema-only admissions criteria were developed based on a large body of evidence indicating that MUAC better identifies children with high mortality risk. However, because MUAC and WHZ identify distinct groups of children, those that would have qualified based on low WHZ are no longer eligible [21]. Our analysis of SAM admissions under the standard treatment protocol found that 98.4% of children would have qualified for admission using the adapted admission criteria based only on MUAC and edema. However, the observed overlap is substantially greater than other estimates and should be considered with caution because it is not nationally representative and potentially influenced by the limited number of locations and organizations included in the analysis. These findings suggest that other factors were more likely drivers of the decreased CMAM caseload during COVID-19. The populations of children admitted to OTP would have been largely similar if standard admission criteria were in place. While raising the MUAC threshold has been suggested as an alternative to combining WHZ and MUAC admissions criteria and is supported by a substantial evidence base, [28, 32] in South Sudan, investments in identifying children with acute malnutrition and maintaining and improving program coverage may be more worthwhile.

It is also worth noting that there are several possible explanations for the relatively large proportion of children admitted based only on WHZ with unknown outcomes. Unknown classification in this group is more likely given the greater likelihood of missing anthropometric data at exit as WHZ is harder to collect than MUAC. Moreover, program quality differences/biases may have impacted outcomes since WHZ only admissions were identified at only half of CMAM sites. Shorter LoS in this group also suggests that children exited the program earlier, possibly as transfers to inpatient care due to complications.

### Reduced visit frequency and modified dosing

Reduced frequency of follow-up visits lessens crowding of CMAM sites, enables social distancing, and reduces risks and burden of travel for caretakers, potentially improving service access and uptake. Evidence regarding reduced visit frequency is limited, but initial evidence suggests that weight gain is adequate with a reduced frequency of follow-up visits. ^21^ In a study of COVID-19 CMAM adaptations, reduced visit frequency was among the most common adaptation. While caregivers were appreciative of the reduced travel burden, particularly given movement restrictions, previously identified concerns, including storing and managing larger quantities of ready-to-use foods and increased sales, were observed, and participants indicated that increased visit frequency would be preferable. Potential benefits of reduced visit frequency that may have contributed to improved recovery rates include reduced travel burden for caretakers and a reduction in resources needed per child at the service provider level that can translate to the ability to manage a larger caseload and/or more time for screening and outreach [33, 34].

Modified dosing intended to optimize RUF dosages for recovery, where ration size is larger in relation to child weight early in treatment, can improve program efficiency, cost-effectiveness, and coverage. Various approaches to modified dosing have been tested, and strong evidence prior to the pandemic likely contributed to widespread adoption as a COVID-19 prevention and control measure. There have been five randomized control trials of modified dosing, all of which demonstrated non-inferior recovery rates and no difference in LoS, though findings were mixed with respect to average weight gain, which is likely a result of different dosing regimens.^21^ In the context of COVID-19, modified dosing enabled CMAM continuity during the suspension of weight measurements while simplifying and streamlining service provision for staff. Implementers expressed some concerns about the negative impact on progress. They indicated a potential return to standard dosing post-pandemic, while some caregivers expressed dissatisfaction with the modified dosage (which may be related to household food insecurity) [35].

In this study, recovery rates > 95% were observed for protocols that included modified dosing, both with and without changes in visit frequency in 2021, which compares to an 88% recovery rate under the standard protocol in 2019. The adjusted odds of recovery, which isolate program effects by accounting for differences in recovery by age, sex, and MUAC admission, were also significantly greater for all three adapted protocols compared to the standard protocol. These findings are similar to national-level trends in South Sudan, where the median OTP recovery rate was 92.0% pre-COVID compared to 95.7% during and after COVID-19; decreases were observed for non-recovered and default program exits, while mortality remained constant across the two time periods.^30^ Numerous contextual factors may have contributed to improved recovery rates observed in the present study including conflict, flooding and other climate-related events, and other barriers to children accessing treatment sites. Nevertheless, findings from South Sudan align with a recent study of outcomes following COVID-19 adaptations to acute malnutrition programs in Uganda, Ethiopia, and Somalia, which found consistent recovery rates that were well within Sphere standards, refuting the expectation of recovery declines with reduced COVID-19-related protocol adaptations [36].

### Recommendations for future research

Analysis of existing CMAM program data from other countries is needed to determine if similar patterns to those seen in this study were observed elsewhere and is a critical next step for informing future guidance on the treatment of children with SAM. Additionally, future research utilizing primary data collection would be beneficial to obtain more precise data for children’s age, WHZ, and treatment outcomes (e.g., reducing the number of “unknown” outcomes). Comprehensive analyses that account for individual child characteristics as well as potential contextual factors influencing recovery and LoS are also needed.

### Limitations

A primary limitation of this study was data availability. A limited number of NGOs store individual-level CMAM data electronically, which restricted the amount of data that could be included due to the time and cost requirements of data entry. As a result, the analysis includes data from a relatively small number of states and NGOs and is not representative of CMAM programs nationally. The number of records each NGO contributed varied widely, leading to a small number of organizations contributing the majority of records; this could result in bias if program outcomes in these organizations differed from those of other NGOs. Additionally, numerous concerns with data quality resulted in the exclusion of many records. In particular, most NGOs did not provide data on children with moderate acute malnutrition, precluding a similar analysis for supplementary feeding programs. Among SAM children, exit data were often incomplete, with MUAC and/or exit type unrecorded. In addition to reducing the sample size, data quality issues could have resulted in bias, particularly if the distribution of missing data was not random. With regard to the analysis, it was not possible to conduct a concurrent comparison of the standard and adapted protocols, which would have been preferable because the impacts of exogenous factors such as supply chain disruptions or changes in food security on outcomes were more likely to be similar. The adjusted analysis attempts to minimize these impacts by excluding data from 2020 when COVID-19-related disruptions were greatest. In addition, there were a number of other unmeasured confounders such as severity of malnutrition and differing patterns of underlying illness in the different years. Finally, it was not possible to obtain data on relapse rates; thus, the study could not explore if and how relapse rates varied under the different treatment protocols.

## Conclusions

In this analysis of 2019 to 2021 program data from South Sudan, which adjusts for individual characteristics to better isolate the effects of protocol adaptations, we observed that children with SAM had shorter LoS and higher recovery rates under the adapted protocols compared to pre-COVID when standard treatment protocols were in place. Additionally, as less than 2% of children admitted under the standard treatment protocol would be ineligible based on MUAC, this study provides compelling evidence that admission criteria modification did not impact CMAM program caseload and that few children were excluded when WHZ entry/exit criteria were suspended.

Differences in LoS when different adaptations were in place suggest that protocol adaptations may lead to shorter recovery and program enrollment times. Given that the population of children eligible for treatment was largely similar, the fact that recovery rates and odds of recovery are improved indicates that the other adaptations, notably reduced visit frequency and simplified dosing, likely contributed to improved recovery rates. While these findings are unexpected in many ways, they suggest that in South Sudan, reverting to standard protocols may not lead to significant improvements in program outcomes, and that maintaining adaptations may be better operationally and in terms of treatment outcomes.

## Data Availability

The datasets analyzed during the current study are available in the Humanitarian Data Exchange and [upon acceptance] can be accessed at https://data.humdata.org/dataset/cdc-ssd-individual-cmam.

## Abbreviations

AOR: Adjusted odds ratio
CDC: US Centers for Disease Control and Prevention
CI: Confidence interval
CMAM: Community Management of Acute Malnutrition
GAM: Global acute malnutrition
ICC: Intraclass correlation coefficient
IQR: Interquartile range
KIIs: Key informant interview
LoS: Length of stay
MUAC: Mid-upper arm circumference
NGO: Non-governmental organization
OR: Odds ratio
OTP: Outpatient Therapeutic Feeding Program
RUTF: Ready-to-use therapeutic food
SAM: Severe acute malnutrition
WHZ: Weight-for-height *Z*-score
